# *Marinobacter salarius* sp. nov. and *Marinobacter similis* sp. nov., Isolated from Sea Water

**DOI:** 10.1371/journal.pone.0106514

**Published:** 2014-09-08

**Authors:** Hooi Jun Ng, Mario López-Pérez, Hayden K. Webb, Daniela Gomez, Tomoo Sawabe, Jason Ryan, Mikhail Vyssotski, Chantal Bizet, François Malherbe, Valery V. Mikhailov, Russell J. Crawford, Elena P. Ivanova

**Affiliations:** 1 Faculty of Science, Engineering and Technology, Swinburne University of Technology, Hawthorn, Victoria, Australia; 2 Universidad Miguel Hernandez, Apartado, San Juan de Alicante, Spain; 3 Laboratory of Microbiology, Faculty of Fisheries, Hokkaido University, Minato-cho, Hakodate, Japan; 4 Callaghan Innovation, Lower Hutt, Wellington, New Zealand; 5 Collection de 1’Institut Pasteur, Institut Pasteur, Paris, France; 6 G.B. Elyakov Pacific Institute of Bioorganic Chemistry of the Far-Eastern Branch of the Russian Academy of Sciences, Vladivostok, Primorski Krai, Russian Federation; Universidade Federal do Rio de Janeiro, Brazil

## Abstract

Two non-pigmented, motile, Gram-negative marine bacteria designated R9SW1^T^ and A3d10^T^ were isolated from sea water samples collected from Chazhma Bay, Gulf of Peter the Great, Sea of Japan, Pacific Ocean, Russia and St. Kilda Beach, Port Phillip Bay, the Tasman Sea, Pacific Ocean, respectively. Both organisms were found to grow between 4°C and 40°C, between pH 6 to 9, and are moderately halophilic, tolerating up to 20% (w/v) NaCl. Both strains were found to be able to degrade Tween 40 and 80, but only strain R9SW1^T^ was found to be able to degrade starch. The major fatty acids were characteristic for the genus *Marinobacter* including C_16:0_, C_16:1_ *ω*7c, C_18:1_ *ω*9c and C_18:1_ *ω*7c. The G+C content of the DNA for strains R9SW1^T^ and A3d10^T^ were determined to be 57.1 mol% and 57.6 mol%, respectively. The two new strains share 97.6% of their 16S rRNA gene sequences, with 82.3% similarity in the average nucleotide identity (ANI), 19.8% similarity in the *in silico* genome-to-genome distance (GGD), 68.1% similarity in the average amino acid identity (AAI) of all conserved protein-coding genes, and 31 of the Karlin's genomic signature dissimilarity. A phylogenetic analysis showed that R9SW1^T^ clusters with *M. algicola* DG893^T^ sharing 99.40%, and A3d10^T^ clusters with *M. sediminum* R65^T^ sharing 99.53% of 16S rRNA gene sequence similarities. The results of the genomic and polyphasic taxonomic study, including genomic, genetic, phenotypic, chemotaxonomic and phylogenetic analyses based on the 16S rRNA, *gyrB* and *rpoD* gene sequence similarities, the analysis of the protein profiles generated using MALDI-TOF mass spectrometry, and DNA-DNA relatedness data, indicated that strains R9SW1^T^ and A3d10^T^ represent two novel species of the genus *Marinobacter*. The names *Marinobacter salarius* sp. nov., with the type strain R9SW1^T^ ( =  LMG 27497^T^  =  JCM 19399^T^  =  CIP 110588^T^  =  KMM 7502^T^) and *Marinobacter similis* sp. nov., with the type strain A3d10^T^ ( =  JCM 19398^T^  =  CIP 110589^T^  =  KMM 7501^T^), are proposed.

## Introduction

The genus *Marinobacter* (family *Alteromonadaceae*, order *Alteromonadales*, class *Gammaproteobacteria*) was created by Gauthier et al. for a hydrocarbon degrading bacterium. At the time of writing, the genus comprises 33 validly described species, http://www.bacterio.net/marinobacter.html [1], which accommodates Gram-negative, chemoheterotrophic and halophilic, rod-shaped bacteria [2], [3]. The important role played by *Marinobacter* spp. in metabolizing hydrocarbons has long been noted, with *M. hydrocarbonoclasticus* [2], *M. aquaeolei* [4], [5], *M. maritimus* [6], and *M. algicola* [7] having been characterized as being able to utilise aromatic and aliphatic hydrocarbons as their sole carbon and energy sources. It was also shown that bacteria of the genus *Marinobacter* are one of the dominant bacterial community groups constantly recovered from hydrocarbon polluted sites [8]–[10]. For example, it was recently demonstrated that *M. vinifirmus* was able to effectively degrade toluene, benzene, ethylbenzene, and *p*-xylene [11].

The objectives of this study were to classify two newly isolated marine bacteria; strain R9SW1^T^, which was derived from a water sample collected from Chazhma Bay (Gulf of Peter the Great, Sea of Japan, Pacific Ocean) during taxonomic studies of microbial communities developed in sea water contaminated by radionuclides [12]; and strain A3d10^T^, which was isolated from Port Philip Bay (the Tasman Sea, Pacific Ocean) during the course of polymer biodegradation studies [13]. The comparative taxonomic investigations of these bacteria, together with their close relatives, revealed their distinct taxonomic standing. This suggests that strain R9SW1^T^ and strain A3d10^T^ represent two novel species of the genus *Marinobacter*.

## Materials and Methods

### Isolation procedures, bacterial strains, and growth conditions

Strain R9SW1^T^ was isolated from a sea water sample collected from Chazhma Bay in the Sea of Japan, Pacific Ocean, in 2000. Water sample collection was within the research program funded by the Federal Agency for Science of the Ministry of Education and Science of the Russian Federation, grant 2–2.16 and by the Russian Foundation for Basic Research and grant ‘Molecular and Cell Biology’ from the Presidium of the Russian Academy of Sciences, grant 02-04-48211". The specific location of the studies (GPS coordinates) was 42°53′38″ N 132°22′02″ E. The permit issued by the Department of Marine Expeditions, Ministry of Education and Science of the Russian Federation. Strain A3d10^T^ was isolated from a sea water sample collected one metre below the water surface in Port Philip Bay, the Tasman Sea, Pacific Ocean, in 2008. Sea water collected from St Kilda Beach which is a publicly accessible beach area in Melbourne, not part of any protected area of land or sea. Furthermore, the field studies did not involve endangered or protected species. The specific location of the studies (GPS coordinates) was 37°51′50″S 144°58′55″E. The sample handling and isolation procedures used were as previously described [12], [13]. Samples were plated on marine agar 2216 (BD, USA) and incubated aerobically at approximately 22–25°C for 5, 7 or 10 days. The isolation and purification procedure has been described elsewhere [14], [15]. Ten type strains of the *Marinobacter* species were obtained from various culture collections and used as the reference strains; *M. lipolyticus* CIP 107627^T^, *M. gudaonensis* CIP 109534^T^, *M. adhaerens* CIP 110141^T^, *M. salsuginis* CIP 109893^T^ and *M. flavimaris* CIP 108615^T^ were obtained from Collection de l’Institut Pasteur (CIP) culture collection, *M. algicola* LMG 23835^T^, *M. guineae* LMG 24048^T^ and *M. sediminum* LMG 23833^T^ were obtained from The Belgian Co-ordinated Collections of Micro-organisms (BCCM/LMG), *M. goseongensis* KCTC 12515^T^ was obtained from Korean Collection for Type Cultures (KCTC) and *M. xestospongiae* JCM 17469^T^ was obtained from RIKEN BRC-Japan Collection of Microorganisms (JCM). The type species of the genus, *M. hydrocarbonoclasticus* SP. 17^T^ was kindly provided by Dr. Stan-Lotter. Strains were stored at −80°C in marine broth 2216 (BD, USA) that had been supplemented with 20% (v/v) glycerol.

### 16S rDNA, *gyrB*, *rpoD* sequencing and phylogenetic analysis

Genomic DNAs were isolated using a Wizard Genomic DNA Purification Kit (Promega, USA) according to the manufacturer's specifications. The 16S rRNA gene sequences for strains R9SW1^T^ and A3d10^T^ were extracted from the whole genome sequences [16] while *gyrB* and *rpoD* genes were amplified using primers (see Supporting Information, Table S1 in File S1) that have been previously described [17], [18]. The 16S rRNA gene sequences of validly described *Marinobacter* species were retrieved from GenBank and aligned using the CLUSTAL W program [19]. Evolutionary phylogenetic trees were constructed using the neighbour-joining (NJ) [20], maximum-likelihood (ML) [21] and maximum-parsimony (MP) [22] algorithms. Genetic distances were calculated using Kimura's two-parameter model [23] by using the MEGA 5 software [24]. The GenBank/EMBL/DDBJ accession numbers of 16S rRNA gene, *gyrB*, *rpoD* and whole genome sequences were presented as in Table 1.

**Table 1:** GenBank/EMBL/DDBJ accession numbers of 16S rDNA, *gyrB*, *rpoD* and whole genome sequences for strains R9SW1^T^, A3d10^T^ and phylogenetically related type strains and type species of the genus *Marinobacter.*

Species name	GenBank/EMBL/DDBJ accession numbers			
	16S rDNA	*gyrB*	*rpoD*	whole genome
Strain R9SW1^T^	KJ547705	KF811464	KF811478	CP007152
Strain A3d10^T^	KJ547704	KF811465	KF811471	CP007151
*M. algicola* LMG 23835^T^	AY258110*	KF811463	KF811474	-
*M. sediminum* LMG 23833^T^	AJ609270*	KF811466	KF811477	-
*M. adhaerens* CIP 110141^T^	AY241552*	KF811467	KF811473	NC_017506*
*M. flavimaris* CIP 108615^T^	AY517632*	KF811468	KF811475	-
*M. salsuginis* CIP 109893^T^	EF028328*	KF811469	KF811476	-
*M. hydrocarbonoclasticus* SP.17^T^	X67022*	KF811470	KF811472	NC_017067*

*Accession numbers from previous publications.

### MALDI-TOF MS analysis

The sample preparation and MALDI-TOF MS analysis was carried out according to the techniques described elsewhere [25]. Briefly, 5 µL of the cultures grown overnight were transferred into microcentrifuge tubes and subjected to ethanol and formic acid protein extraction. One µL aliquots of the supernatant were transferred onto the MALDI target plate and air dried at room temperature, followed by the addition of 1 µL of matrix solution, then air dried. Samples were then subjected to analysis using a Microflex MALDI-TOF mass spectrometer (Bruker Daltonik GmbH, Leipzig, Germany) equipped with a 60 Hz nitrogen laser. Spectra were recorded in the positive linear mode for the mass range of 2,000 to 20,000 Da at the maximum laser frequency. The raw spectra were then analysed using the MALDI Biotyper 3.0 software package (Bruker Daltonik GmbH, Bremen, Germany) under the default settings. Measurements were performed via the automatic mode, without any user intervention.

### GC content and DNA-DNA hybridization

The GC content of strains R9SW1^T^ and A3d10^T^ was calculated on the basis of their whole genome sequences [16], [26], and these have been deposited at GenBank/EMBL/DDBJ under the accession number of CP007152 and CP007151, respectively. The DNA-DNA hybridizations between strain R9SW1^T^ and *M. algicola* LMG 23835^T^, and strain A3d10^T^ and *M. sediminum* LMG 23833^T^ were performed by the Deutsche Sammlung von Mikroorganismen und Zellkulturen (DSMZ) identification service, where cells were initially disrupted using a Constant Systems TS 0.75 KW (IUL Instruments, Germany), followed by purification of the extracted DNA in the crude lysate form by chromatography on hydroxyapatite as described by Cashion et. al. (1977) [27]. DNA-DNA hybridization was carried out in duplicate using a 2× saline sodium citrate (SSC) buffer with 5% formamide as described by De Ley et al. [28], with consideration of the modifications described by Huss et. al. (1983) [29], using a model Cary 100 Bio UV/VIS-spectrophotometer equipped with a Peltier-thermostatted 6×6 multi-cell changer and a temperature controller with an *in-situ* temperature probe (Varian).

### Genome comparison and genomic signatures analyses

Complete genome sequences for only two validly described species of *Marinobacter*, *M. hydrocarbonoclasticus* ATCC 49840^T^ [30] and *M. adhaerens* HP15^T^ [31], which have previously been assembled, were used in this study for genomic analysis. The fully sequenced and assembled genomes of both these species were retrieved from GenBank, and compared to those of R9SW1^T^ and A3d10^T^. Genome comparison between strains R9SW1^T^, A3d10^T^, *M. adhaerens* HP15^T^ and *M. hydrocarbonoclasticus* ATCC 49840^T^ was carried out using reciprocal BLAST analysis, according to the method described by Goris et al. [32]. A map of the percentage identity between each of *M. adhaerens* HP15^T^, R9SW1^T^ and A3d10^T^ to the type species was generated using the BLAST Ring Image Generator (BRIG) software [33]. The in-silico genome-to-genome distance (GGD) between the four strains was also calculated using the genome-to-genome distance calculator 2.0 (GGDC) provided by DSMZ, http://ggdc.dsmz.de [34], [35]. The average amino acid identity (AAI) of all conserved protein-coding genes was calculated as previously described [36]. The conserved genes between a pair of genomes were determined by whole-genome pairwise sequence comparison using the BLAST algorithm release 2.2.5 [37] using a minimum cut-off of 40% identity and 70% of the length of the query gene. The difference in genome signature between two individual sequences is expressed in terms of the Karlin's genomic signature dissimilarity (δ*), which was calculated by dividing the genomic dinucleotide frequencies with the corresponding mononucleotide content using the equation described by Karlin et al. [38]. Phylogenomic relationship between the four strains were also elucidated using Mauve multiple alignment software (v2.3.1) [39] and ClonalFrame software v1.2 [40], with *Alteromonas* sp. DE [41] used as an outgroup.

Genotype to phenotype analyses of a few distinctive phenotypes were also carried using the whole genome sequences of strains R9SW1^T^, A3d10^T^, *M. hydrocarbonoclasticus* ATCC 49840^T^ and *M. adhaerens* HP15^T^ using the methods as previously described [42].

### Physiological and biochemical analysis

Six reference type strains, along with strains R9SW1^T^ and A3d10^T^, were used for the phenotypic and biochemical tests (Table 2). The cell morphology and motility were determined using scanning electron and light microscopies. Gram stain reaction, catalase (5% H_2_O_2_) and starch hydrolysis analyses were performed according to the method described by Smibert and Krieg (1994) [43]. Determination of the oxidase activity was performed using Bactident oxidase strips (Merck Millipore, Germany). The capacity of the strains to oxidize and to ferment _D_-glucose and lactose was carried out according to the method described by Smibert and Krieg (1994) [43], using a modified semi-solid medium containing: 9.4 g L^−1^ O/F medium (Oxoid, UK), 20 g L^−1^ Sea Salt (Sigma-Aldrich, USA) and 1% carbohydrate. The strains were incubated at 30°C and the results were obtained after 48 hours. The temperature and pH tolerance ranges were determined via marine agar growth tests subjected to different temperature (4, 10, 15, 20, 25, 30, 37, 40, 45 and 50°C) and pH (4, 6, 7, 8, 9 and 11, adjusting the pH with HCl and NaOH) conditions. The NaCl tolerance was determined using different concentrations of NaCl (0, 0.5, 1, 3, 6, 10, 15, 20 and 25%) in modified salinity agar (SA) containing: 5 g L^−1^ peptone, 1 g L^−1^ yeast extract, 0.1 g L^−1^ ferric citrate, 3.24 g L^−1^ magnesium sulphate (MgSO_4_), 0.55 g L^−1^ dipotassium phosphate (K_2_HPO_4_), 15 g L^−1^ agar, and the respective NaCl concentration, each at a pH of 7.6±0.2. Plates were incubated under optimal temperature conditions and the results were recorded daily for 7 days.

**Table 2:** Differential characteristics between strains R9SW1^T^, A3d10^T^, their close phylogenetic neighbors and type species of the genus *Marinobacter*.

Characteristics	1	2	3	4	5	6	7	8	9	10	11	12	13
Cell length (µm)	1.9–3.2	1.6–2.5	1.3–2.1	1.8–2.5	2.0–4.0	1.7–2.4	1.5–3.0	2.5–3.5	1.2–1.8	1.6–2.0	2.0–2.5	1.4–4.0	2.0–3.0
Cell width (µm)	0.40–0.72	0.45–0.55	0.40–0.45	0.3–0.4	1.0	0.6–0.8	0.6–0.9	0.3–0.5	0.3–0.5	0.5–0.8	0.6–0.8	0.4	0.3–0.6
Growth temperature (°C)	4–40	5–40	4–40	4–42	10–45	4–45	4–45	15–40	10–45	10–37	15–42	4–42	10–45
pH range	6–9	5–10	6–9	ND	6.5–9.5	5.5–10.0	>5.5	5.0–10.0	6.0–9.5	5.3–9.3	5.0–10.0	5.0–9.5	6–9.5
Salinity range (%, w/v)	0.5–20	1–12	0.5–20	0.5–18	1–20	0.5–20	1–20	1–15	0–15	1–25	0.5–6.0	1–15	1–20
Nitrate reduction	-	+ (-)	+	+	+	-	+	-	+	ND	+	+	+
Nitrite reduction	-	+ (-)	-	+ (-)	- (+)	-	-	-	-	ND	-	+	- (+)
Hydrolysis of starch	+	+	-	-	-	-	-	-	+	-	-	-	-
Indole production	-	-	-	-	-	-	-	-	ND	ND	+	-	-
Fermentation of:													
_D_-Glucose	-	-	w	w	-	w	-	ND	ND	ND	+	+	-
Lactose	-	-	-	-	-	w	-	ND	ND	ND	ND	ND	-
Utilisation of:													
Glycogen	+	+	+	-	-	-	-	+	ND	-	ND	ND	-
Mono-methyl-succinate	+	-	+	+ (-)	+	+	w	-	ND	-	ND	ND	+
*γ*-Hydroxy-butyric acid	+	+	+	-	-	w (+)	w	-	ND	-	ND	ND	-
Succinic acid	+	- (+)	-	-	+	w	- (+)	-	+	-	+	ND	+
_L_-Glutamic acid	+	+	+	w (-)	+	+	+ (-)	-	-	-	+	ND	+
_L_-Phenylalanine	-	+	-	-	- (+)	-	-	-	ND	-	ND	ND	-
_L_-Serine	+	-	-	- (+)	-	-	-	-	ND	-	ND	ND	-
Glycerol	+	- (+)	-	-	w (+)	+ (-)	-	-	+	-	+	+	-
DNA G+C content (mol%)	57.1	55.0	57.6	56.5	55.9	56.9	58.0	57.0	57.9	ND	57.1	57.1	52.7

Strains: 1, strain R9SW1^T^; 2, *M. algicola* LMG 23835^T^; 3, strain A3d10^T^; 4, *M. sediminum* LMG 23833^T^; 5, *M. salsuginis* CIP 109893^T^; 6, *M. adhaerens* CIP 110141^T^; 7, *M. flavimaris* CIP 108615^T^; 8, *M. lipolyticus* SM19^T^; 9, *M. gudaonensis* SL014B61A^T^; 10, *M. goseongensis* En6^T^; 11, *M. xestospongiae* UST090418-1611^T^; 12, *M. guineae* M3B^T^; 13, *M. hydrocarbonoclasticus* SP.17^T^.

Data for nitrate and nitrite reduction, starch hydrolysis, fermentation, indole and acid production, organic substrates utilisation, and enzyme activities for strains R9SW1^T^, *M. algicola* LMG 23835^T^, A3d10^T^, *M. sediminum* LMG 23833^T^, *M. salsuginis* CIP 109893^T^, *M. adhaerens* CIP 110141^T^, *M. flavimaris* CIP 108615^T^ and *M. hydrocarbonoclasticus* SP. 17^T^ are from this study. The data in brackets are from previously published work [2], [7], [62]–[70].

+, Positive; -, Negative; w, Weak reaction; ND, No Data available.

The susceptibility of the bacteria to antibiotics was tested using modified media containing: 21 g L^−1^ Mueller-Hinton medium (Oxoid, UK); 7.5% Sea salt and 15 g L^−1^ bacteriological agar (Agar No. 1, Oxoid, UK). The antibiotics tested were penicillin G (10 µg), chloramphenicol (30 µg), streptomycin (10 µg), tetracycline (30 µg), ampicillin (10 µg) and oxacillin (1 µg). The strains were incubated under optimal temperature conditions and results were obtained after 24 hours of incubation.

The ability of the strains to oxidise a range of organic substrates was investigated using a 96-well Biolog GN2 microplate (Biolog, USA), in triplicate. Inoculates were prepared by suspending culture that had been grown overnight into 3% (w/v) saline solution, then adjusting the density of the suspension to McFarland standard no. 1, followed by pipetting 150 µL aliquots of the suspension into each well. All the plates were incubated at 30°C and results were manually obtained after 24 h and 48 h. Enzymatic tests were performed using API ZYM test strips (bioMérieux, France) in two individual experiments. Inoculations were prepared by suspending culture that had been grown overnight into 3% (w/v) saline solution and adjusting the density to McFarland standard no. 5. A Microbact 24E Gram-negative identification system (Oxoid, UK) was also used to test other biochemical reactions, namely: lysine and ornithine decarboxylase; H_2_S production; glucose, mannitol and xylose fermentation; hydrolysis of o-nitrophenyl-*β*-_D_-galactopyranoside (ONPG); indole production; urea hydrolysis; acetoin production (Voges-Proskaüer reaction); citrate utilisation; production of indolepyruvate; gelatin liquefaction; malonate inhibition; inositol, sorbitol, rhamnose, sucrose, lactose, arabinose, adonitol, raffinose and salicin fermentation; and arginine dihydrolase. All tests were carried out according to the manufacturer's specifications unless otherwise stated.

### Fatty acids analysis

Fatty acid (FA) methyl esters were prepared as described elsewhere [44]. The resulting fatty acid methyl esters were analysed using a Shimadzu GC-14A gas chromatograph with a flame ionization detector, using both a nonpolar SPB-5 fused-silica column (30 m×0.25 mm i.d.) at 210°C and a polar Supelcowax-10 fused-silica column (30 m×0.25 mm i.d.) at 200°C.

## Results and Discussion

**Figure 1:**
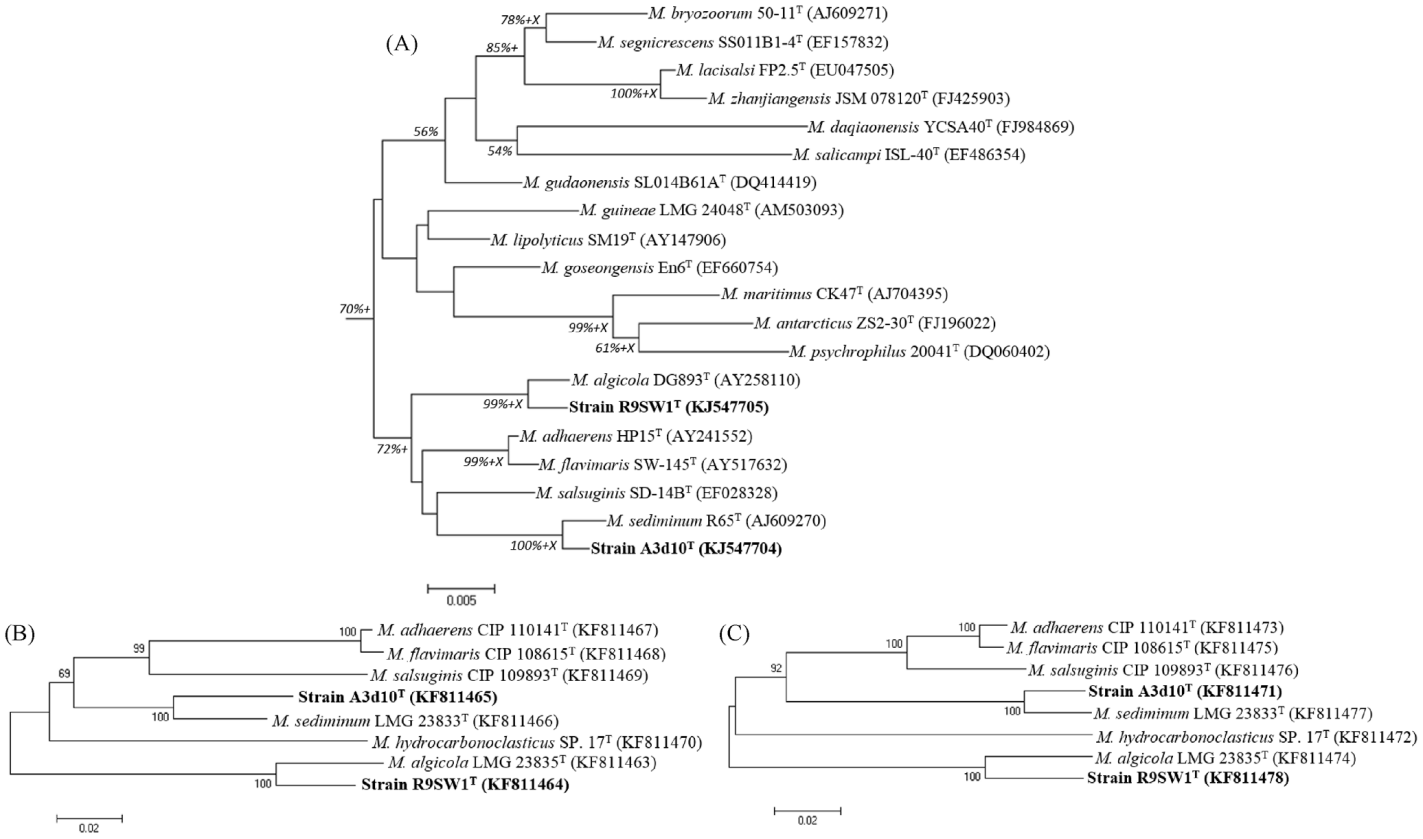
Neighbour-joining phylogenetic tree showing the taxonomic position of strains R9SW1^T^ and A3d10^T^ according to their (A) 16S rRNA, (B) *gyrB* and (C) *rpoD* gene sequences. Numbers at branching points are percentage bootstrap values based on 1000 replications, with only values above 50% are shown. Scale bar represents 0.005/0.02 substitutions per nucleotide position. The Maximum-likelihood (ML) and maximum Parsimony (MP) algorithms were also used for tree construction, where branches in agreement with ML and MP methods were marked with *+* and *X* respectively.

Analysis of the complete 16S rRNA gene sequences of strains R9SW1^T^ and A3d10^T^ revealed that both strains are grouped with species of the genus *Marinobacter*, with the sequence similarity between strains R9SW1^T^, A3d10^T^ and all validly described *Marinobacter* species being in the range of 93.84–99.40% and 93.91–99.53%, respectively. The two new strains, R9SW1^T^ and A3d10^T^ shared 97.6% of their 16S rRNA gene sequences, however, phylogenetic analysis showed that they cluster separately forming two different clusters, one with *M. algicola* DG893^T^ and another with *M. sediminum* R65^T^, where both clusters were supported by the bootstrap value of 99% and 100% in both the NJ and ML methods (Figure 1A and Figure S1 in File S1). The highest 16S rRNA gene sequence similarity between strain R9SW1^T^ and *M. algicola* DG893^T^ was found to be 99.40% (*M. algicola* DG893^T^), whilst strain A3d10^T^ displays the highest 16S rRNA gene sequence similarity with *M. sediminum* R65^T^ (99.53%).

Due to the high 16S rRNA gene sequence similarity between strains R9SW1^T^ and *M. algicola* DG893^T^, and between A3d10^T^ and *M. sediminum* R65^T^, an extended phylogenetic analysis based on *gyrB* and *rpoD* genes was carried out. The use of housekeeping genes in phylogenetic analysis can be beneficial, in that it overcomes the possibility of the presence of nucleotide polymorphisms in the 16S rRNA gene [45], [46]. Two genes, *gyrB* and *rpoD*, were selected, since they have been previously reported to be excellent marker genes and sufficient for the identification and classification of various groups of microorganism [25], [47]–[49]. *M. sediminum* LMG 23833^T^, *M. salsuginis* CIP 109893^T^, *M. algicola* LMG 23835^T^, *M. adhaerens* CIP 110141^T^, and *M. flavimaris* CIP 108615^T^ were selected, as they are phylogenetically close to strains R9SW1^T^ and A3d10^T^ according to their 16S rRNA gene sequences. *M. hydrocarbonoclasticus* SP.17^T^ was also included as representing the type species of the genus. A phylogenetic analysis of the *gyrB* and *rpoD* gene sequence similarities reconfirmed the clustering of strain R9SW1^T^ with *M. algicola* LMG 23835^T^, and strain A3d10^T^ with *M. sediminum* LMG 23833^T^, both of which were supported with 100% bootstrap values (Figure 1(B) and (C)). The *gyrB* and *rpoD* sequence similarities for strains R9SW1^T^, A3d10^T^ and their phylogenetically related species was also determined and found to be in the range of 77.8–94.3% (R9SW1^T^, *gyrB*), 80.0–93.5% (A3d10^T^, *gyrB*), and 78.6–93.8% (R9SW1^T^, *rpoD*), 78.6–96.2% (A3d10^T^, *rpoD*), respectively (Table 3). The gene sequence similarity for *gyrB* and *rpoD* between the previously described sister species of *Marinobacter*, *i.e*., *M. adhaerens* CIP 110141^T^ and *M. flavimaris* CIP 108615^T^ was found to be 99.0% and 98.4% respectively (Table 3), which is higher than that found for strains R9SW1^T^, A3d10^T^ and their respective closest phylogenetic relatives. The sequence similarities of the *gyrB* gene of 94.3% and 93.5% for strains R9SW1^T^, A3d10^T^ with their closest relatives were also lower than the previously proposed *gyrB* sequence similarity cut-off value of 98.95% for genus *Amycolatopsis* [50] and 98.22% for genus *Kribbella* [51]. Also, the data reported for the two *Vibrio* species, *V. gigantis* LGP 13^T^ and *V. crassostreae* LGP 7^T^, were 98% for *gyrB* and 97% for *rpoD* [52], which again showed higher similarity values than the *gyrB* and *rpoD* sequence similarities of strains R9SW1^T^, A3d10^T^ and their closest relatives. The sequence similarities for *gyrB* and *rpoD* between strains R9SW1^T^ and A3d10^T^ were significantly lower than the values mentioned above, *i.e*., 81.6% for *gyrB* and 78.2% for *rpoD*, suggesting distinct standing of new strains on the species level.

**Table 3:** The *gyrB* and *rpoD* gene sequence similarities of strains R9SW1^T^, A3d10^T^ and phylogenetically related type strains and type species of the genus *Marinobacter*.

	Similarity of *gyrB*/*rpoD* genes (%)							
	1	2	3	4	5	6	7	8
1. *M. adhaerens* CIP 110141^T^	100/100							
2. *M. algicola* LMG 23835^T^	78.0/81.2	100/100						
3. *M. flavimaris* CIP 108615^T^	99.0/98.4	77.8/81.0	100/100					
4. *M. hydrocarbonoclasticus* SP. 17^T^	80.7/81.7	78.2/77.0	80.0/81.5	100/100				
5. *M. salsuginis* CIP 109893^T^	86.5/93.4	76.2/80.3	86.1/93.5	80.8/80.0	100/100			
6. *M. sediminum* LMG 23833^T^	84.1/83.5	80.3/77.8	83.8/84.2	83.6/78.7	85.8/84.0	100/100		
7. *Marinobacter* sp. A3d10^T^	83.7/83.8	80.0/78.8	83.6/84.4	82.2/78.6	84.7/84.1	93.5/96.2	100/100	
8. *Marinobacter* sp. R9SW1^T^	78.2/80.5	94.3/93.8	78.2/80.3	77.8/79.5	78.0/80.6	81.9/78.6	81.6/78.2	100/100

**Figure 2:**
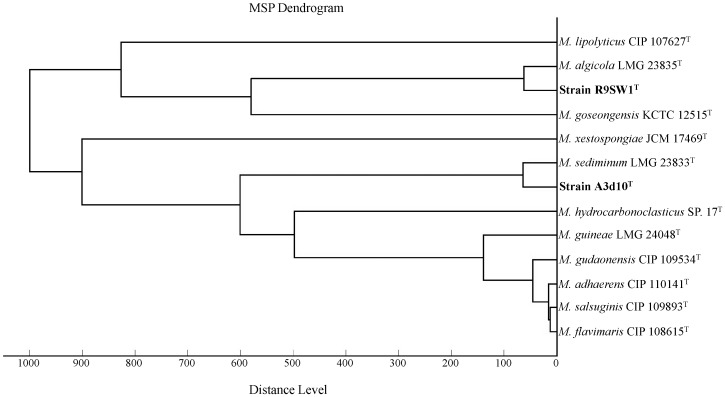
Main spectra library (MSP) dendrogram of MALDI-TOF mass spectral profiles of strains R9SW1^T^, A3d10^T^ and closely related *Marinobacter* species. The dendrogram was generated by MALDI Biotyper 3.0 software with distance is displayed in relative units.

In order to further assess the taxonomic affiliation of the two new bacteria, a comparative analysis of the total protein profiles was performed using MALDI-TOF mass spectrometry (Figure 2). The results are in agreement with the phylogenetic analyses, clearly indicating that strain R9SW1^T^ is clustering with *M. algicola* LMG 23835^T^, and strain A3d10^T^ is clustering with *M. sediminum* LMG 23833^T^ with a critical distance level below 500. As suggested in the previously reported studies, clustering below the distance level of 500 can be considered as reliable clustering [53], [54], which was also in agreement with the recent studies on *Alteromonas* spp., where the clustering within the distance level of 500 was shown to be able to differentiate the closely related *Alteromonas* species [25], [55]. Hence, the results of this study confirmed the confident clustering of the two new isolates within other species of the genus *Marinobacter*. Also, the clusters of both strains R9SW1^T^ and A3d10^T^ with their nearest neighbour were stable, but exceeded the minimum differences between existing species, *e.g*., the distance level between species in both clusters were greater than those within a cluster that contained *M. gudaonensis* CIP 109534^T^, *M. adhaerens* CIP 110141^T^, *M. salsuginis* CIP 109893^T^, and *M. flavimaris* CIP 108615^T^; so does the position of strains R9SW1^T^ and A3d10^T^ resulting in different clusters in the MALDI dendrogram, provide evidence of the distinctive standing of two new bacteria.

**Figure 3:**
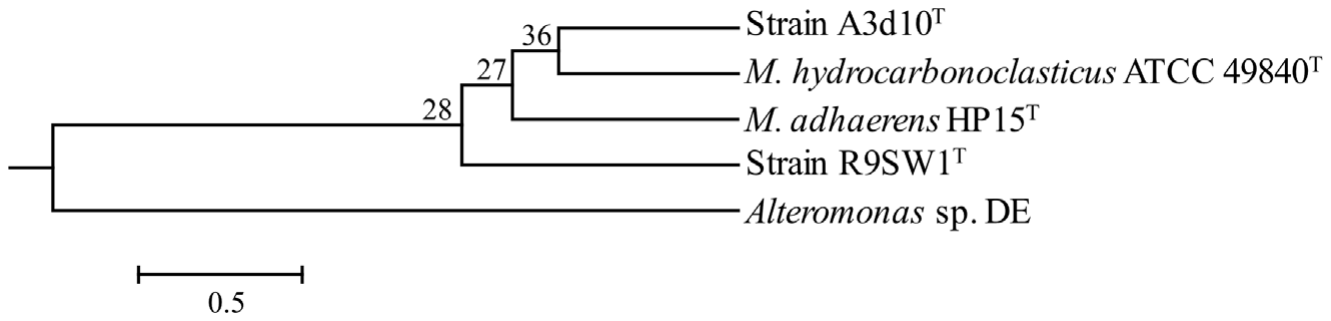
Phylogenomic tree of strains R9SW1^T^, A3d10^T^, *M. hydrocarbonoclasticus* ATCC 49840^T^ and *M. adhaerens* HP15^T^ constructed using concatenated sequence of the core proteome (544,643 bp) of the genomes. *Alteromonas* sp. DE was used as outgroup.

In order to confirm the separate species standing of these two strains, a DNA-DNA hybridization experiment was conducted. DNA-DNA relatedness between strain R9SW1^T^ and *M. algicola* LMG 23835^T^ was found to be 63.05±1.85%, and between strain A3d10^T^ and *M. sediminum* LMG 23833^T^ was found to be 67.60±1.3%. Both of these relatedness values are below the 70% cut-off value generally recommended for species differentiation [56]. Recently, information of whole genome sequences have been recommended to be integrated into bacterial systematics [57]–[59]. In this study, whole genome sequences of strains R9SW1^T^, A3d10^T^, *M. adhaerens* HP15^T^ and *M. hydrocarbonoclasticus* ATCC 49840^T^ were visually compared using BLAST (Figure S2 in File S1) and the average nucleotide identity (ANI), genome-to-genome distance (GGD), average amino acid identity (AAI), and the Karlin's genomic signature dissimilarity (δ*) between the four strains were calculated, the results of which are presented in Table 4. Due to the lack of the availability of the assembled, whole genome sequences for validly named *Marinobacter* species, genomic signatures between strains R9SW1^T^, A3d10^T^ and validly described *Marinobacter* species can only be performed using those of *M. adhaerens* HP15^T^ [31] and *M. hydrocarbonoclasticus* ATCC 49840^T^ [30]. As can be seen from the information presented in Table 4, the ANIs between the four strains were in the range of 82.3–83.3%, which is significantly lower than the suggested threshold range of 95–96% [58], [60]; the GGDs were calculated to be in the range of 19.8–20.7% which is lower than the cur-off value of 70% [61]; the AAI and Karlin signature dissimilarity values for the four strains were in the range of 68.1–77.6% and 31–36 respectively, each of which fall outside the range to be consider as same species [42], [61]; and thus again indicating that strains R9SW1^T^ and A3d10^T^ can be considered as two novel species of the genus *Marinobacter*. The distinct standing of strains R9SW1^T^ and A3d10^T^ can also be confirmed by the phylogenomic relationship analysis using the core proteome of the genomes from the four strains (Figure 3).

**Table 4:** The genomic signatures between strains R9SW1^T^, A3d10^T^, *M. adhaerens* HP15^T^ and *M. hydrocarbonoclasticus* ATCC 49840^T^.

	Genomic signatures			
	1	2	3	4
1. *M. hydrocarbonoclasticus* ATCC 49840^T^		20.1/35	20/31	19.8/32
2. *M. adhaerens* HP15^T^	83.1/74.5		20.2/36	20.7/35
3. Strain R9SW1^T^	82.3/69.5	82.7/72.6		19.8/31
4. Strain A3d10^T^	82.5/72.7	83.3/77.6	82.3/68.1	

Data in the lower triangular corresponds to ANI/AAI (%) and data in the upper triangular corresponds to GGD (%)/Karlin signature.

The major features of the genomes of strains R9SW1^T^ and A3d10^T^ were identified as described elsewhere [16]. Briefly, they are 4,616,532 bp and 3,975,896 bp in size, composed of 99 and 29 contigs, both have 3 rRNAs, and 44 and 46 tRNAs, for strains R9SW1^T^ and A3d10^T^, respectively. The DNA G+C content of strains R9SW1^T^ and A3d10^T^ were found to be 57.1 and 57.6 mol%, respectively (Table 2), the values which are consistent with those of the genus *Marinobacter*.

Both bacteria were found to be Gram-negative, aerobic, motile by means of a single flagellum and rod-shaped with the size of 1.9–3.2×0.40–0.72 µm for strain R9SW1^T^ and 1.3–2.1×0.40–0.45 µm for strain A3d10^T^ (Figure S3 in File S1). The catalase and oxidase tests were found to be positive, H_2_S and indole tests were found to be negative. It can be seen that strain R9SW1^T^ can be clearly differentiated from *M. algicola* LMG 23835^T^ by its inability to reduce nitrate and nitrite, its ability to utilise mono-methyl succinate and _L_-serine, its inability to utilise _L_-phenylalanine and the absence of lipase (C14); while strain A3d10^T^ can be clearly differentiated from *M. sediminum* LMG 23833^T^ by its inability to reduce nitrite, its ability to utilise glycogen, *γ*-hydroxy-butyric acid and _L_-glutamic acid, and its weak activities for valine arylamidase and cystine arylamidase. The major phenotypic difference between strains R9SW1^T^ and A3d10^T^ are nitrate reduction, hydrolysis of starch, fermentation of _D_-glucose, and their utilisation of dextrin, _D_-fructose, maltose, acetic acid, propionic acid, succinic acid, _L_-serine and glycerol. Other phenotypic characteristics which differentiate the two novel strains from each other and their closest phylogenetic neighbours are shown in Table 2, Table S2 in File S1, and in their respective species descriptions. Both strains were found to be sensitive to penicillin G (10 µg), chloramphenicol (30 µg), and ampicillin (10 µg), and resistant to streptomycin (10 µg) and tetracycline (30 µg). The fatty acid composition of strains R9SW1^T^ and A3d10^T^ are shown in Table S3 in File S1, where the predominant fatty acids were identified as being C_16:0_, C_16:1_ *ω*7c, C_18:1_ *ω*9c and C_18:1_ *ω*7c.

The genotype to phenotype analyses were also carried out based on the whole genome sequences of the four strains, the results of which are presented in Table 5. It can be seen that of the results of physiological and biochemical tests match when comparing the *in silico* results, however a few discrepancies are noted. A similar level of deviation previously reported in the case of *Vibrio* species and it was suggested that expression of certain genes may be restricted by stop codon, repressor genes, regulatory proteins, global regulators, genome coverage or sequencing errors [42].

**Table 5:** Comparative identification of phenotypic characteristics based on genomic analysis and physiological and biochemical tests.

	Strain R9SW1^T^		Strain A3d10^T^		*M. hydrocarbonoclasticus* ATCC 49840^T^		*M. adhaerens* HP15^T^	
	*In vitro*	*In silico*	*In vitro*	*n silico*	*In vitro*	*In silico*	*In vitro*	*In silico*
Nitrate reduction	-	+	+	+	+	+	-	-
Nitrite reduction	-	-	-	-	-	+	-	-
Hydrolysis of starch	+	-	-	-	-	-	-	-
Indole production	-	-	-	-	-	-	-	-
D-Glucose	-	-	w	-	-	-	w	-
Lactose	-	-	-	-	-	-	w	-
Glycogen	+	-	+	-	-	+	-	+
Mono-methyl-succinate	+	+	+	+	+	+	+	+
γ-Hydroxy-butyric acid	+	-	+	-	-	-	w	+
Succinic acid	+	+	-	-	+	+	w	+
L-Glutamic acid	+	+	+	+	+	+	+	+
L-Phenylalanine	-	-	-	-	-	-	-	-
L-Serine	+	+	-	+	-	-	-	-
Glycerol	+	+	-	-	-	-	+	-

In summary, the comparative genomic and phylogenetic analysis based on the full-length of 16S rRNA gene sequence similarities, pheno- and chemotaxonomic properties revealed that strains R9SW1^T^ and A3d10^T^ can be affiliated to the genus *Marinobacter*. A further dual-locus sequence analysis based on *gyrB* and *rpoD* gene sequence similarities, the comparative analysis of whole cells protein profiles based on MALDI-TOF mass spectrometry analysis, their phenotypic characteristics and their DNA-DNA hybridization values below 70% confirmed that strains R9SW1^T^ and A3d10^T^ should be classified as two novel species of the genus *Marinobacter* for which the name *Marinobacter salarius* sp. nov. and *Marinobacter similis* sp. nov. are proposed.

## Description of *Marinobacter salarius* sp. nov

### *Marinobacter salarius* (*sa.la*'*ri.us*, L. masc. adj., salarius, of or belonging to salt, pertaining to salt tolerance)

Cells are Gram-negative rods (approximately 1.9–3.2×0.40–0.72 µm). Motile by means of a single polar flagellum. Colonies on marine agar are semi-translucent, non-pigmented, circular to slightly irregular (0.8–1.0 mm) and smooth after 48 hours of incubation. Colonies turn to creamy in colour with increasing incubation time. Growth occurs at 4°C–40°C (optimum, 25°C–30°C), between pH 6–9 (optimum, pH 7.5) and in the presence of 0.5–20% (w/v) NaCl. No growth was observed at 0 or 25% (w/v) NaCl. Catalase and oxidase tests are positive. Starch, Tween 40 and 80 are positive, while nitrate and nitrite reduction are negative. Indole, lysine decarboxylase, ornithine decarboxylase, *β*-galactosidase, tryptophan deaminase, gelatinase, arginine dihydrolase, acetoin, urea and H_2_S are not produced. Acid is not produced from glucose, mannitol, xylose, inositol, sorbitol, rhamnose, sucrose, lactose, arabinose, adonitol, raffinose and salicin. According to API ZYM, strain R9SW1^T^ is positive for alkaline phosphatase, esterase (C4), esterase lipase (C8), leucine arylamidase, valine arylamidase, cystine arylamidase and *N*-acetyl-*β*-glucosaminidase; weakly positive for acid phosphatase, naphthol-AS-BI-phosphohydrolase and *α*-glucosidase; negative for lipase (C14), trypsin, *α*-chymotrypsin, *α*-galactosidase, *β*-galactosidase, *β*-glucuronidase, *β*-glucosidase, *α*-mannosidase and *α*-fucosidase. Positive for the utilization of dextrin, glycogen, _D_-fructose, maltose, methyl-pyruvate, mono-methyl-succinate, acetic acid, *β*-hydroxybutyric acid, *γ*-hydroxybutyric acid, _DL_-lactic acid, propionic acid, succinic acid, _L_-glutamic acid, _L_-proline, _L_-serine and glycerol; weakly positive for the utilization of _L_-alaninamide, _D_-alanine, _L_-alanine and _L_-leucine; negative for the utilization of *α*-cyclodextrin, *N*-acetyl-_D_-galactosamine, *N*-acetyl-_D_-glucosamine, adonitol, _L_-arabinose, _D_-arabitol, _D_-cellobiose, i-erythritol, _L_-fucose, _D_-galactose, gentiobiose, *α*-_D_-glucose, *m*-inositol, *α*-_D_-lactose, lactulose, _D_-mannitol, _D_-mannose, _D_-melibiose, *β*-methyl-_D_-glucoside, _D_-psicose, _D_-raffinose, _L_-rhamnose, _D_-sorbitol, sucrose, _D_-trehalose, turanose, xylitol, *cis*-aconitic acid, citric acid, formic acid, _D_-galactonic acid lactone, _D_-galacturonic acid, _D_-gluconic acid, _D_-glucosaminic acid, _D_-glucuronic acid, *α*-hydroxybutyric acid, *p*-hydroxyphenylacetic acid, itaconic acid, *α*-ketoglutaric acid, *α*-ketobutyric acid, *α*-ketovaleric acid, malonic acid, quinic acid, _D_-saccharic acid, sebacic acid, bromosuccinic acid, succinamic acid, glucuronamide, _L_-alanyl-glycine, _L_-asparagine, _L_-aspartic acid, glycyl-_L_-aspartic acid, glycyl-_L_-glutamic acid, _L_-histidine, hydroxyl-_L_-proline, _L_-ornithine, _L_-phenylalanine, _L_-pyroglutamic acid, _D_-serine, _L_-threonine, _DL_-carnitine, *γ*-aminobutyric acid, urocanic acid, inosine, uridine, thymidine, phenyethylamine, putrescine, 2-aminoethanol, 2,3-butanediol, _DL_-*α*-glycerol, glucose-1-phosphate and glucose-6-phosphate as the sole carbon and energy source. The main cellular fatty acids are C_16:0_, C_16:1_ *ω*7c, C_18:1_ *ω*9c and C_18:1_ *ω*7c. The G+C content of the type strain is 57.1 mol%. The type strain is R9SW1^T^ ( =  LMG 27497^T^  =  JCM 19399^T^  =  CIP 110588^T^  =  KMM 7502^T^), isolated from sea water from Chazhma Bay in the Sea of Japan, Pacific Ocean. The accession number for the whole genome sequence of strain R9SW1^T^ is CP007152.

## Description of *Marinobacter similis* sp. nov

### *Marinobacter similis* (*si*'*mi.lis*, L. masc. adj., similis, like, resembling, similar, pertaining to close similarity with other species)

Cells are Gram-negative rods (approximately 1.3 - 2.1×0.40 - 0.45 µm). Motile by means of a single polar flagellum. Colonies on marine agar are semi-translucent, non-pigmented, circular to slightly irregular (0.5 – 1.0 mm) and smooth after 48 hours of incubation. Colonies turn to creamy in colour with increasing incubation time. Growth occurs at 4°C - 40°C (optimum, 25°C - 30°C), between pH 6 to 9 (optimum, pH 7.5) and in the presence of 0.5–20% (w/v) NaCl. No growth was observed at 0 or 25% w/v NaCl. Catalase and oxidase tests are positive. Tween 40 and 80 are positive, while starch is not. Nitrate is reduced but not nitrite. Indole, lysine decarboxylase, ornithine decarboxylase, *β*-galactosidase, tryptophan deaminase, gelatinase, arginine dihydrolase, acetoin, urea and H_2_S are not produced. Acid is not produced from glucose, mannitol, xylose, inositol, sorbitol, rhamnose, sucrose, lactose, arabinose, adonitol, raffinose and salicin. According to API ZYM, strain A3d10^T^ is positive for alkaline phosphatase, esterase (C4), esterase lipase (C8), leucine arylamidase, naphthol-AS-BI-phosphohydrolase, and *N*-acetyl-*β*-glucosaminidase; weakly positive for lipase (C14), valine arylamidase, cystine arylamidase and acid phosphatase; negative for trypsin, *α*-chymotrypsin, *α*-galactosidase, *β*-galactosidase, *β*-glucuronidase, *β*-glucosidase, *α*-glucosidase, *α*-mannosidase and *α*-fucosidase. Positive for the utilization of, glycogen, methyl-pyruvate, mono-methyl-succinate, *β*-hydroxybutyric acid, *γ*-hydroxybutyric acid, _DL_-lactic acid, _D_-alanine, _L_-alanine, _L_-glutamic acid and _L_-proline; negative for the utilization of *α*-cyclodextrin, dextrin, *N*-acetyl-_D_-galactosamine, *N*-acetyl-_D_-glucosamine, adonitol, _L_-arabinose, _D_-arabitol, _D_-cellobiose, i-erythritol, _D_-fructose, _L_-fucose, _D_-galactose, gentiobiose, *α*-_D_-glucose, *m*-inositol, *α*-_D_-lactose, lactulose, maltose, _D_-mannitol, _D_-mannose, _D_-melibiose, *β*-methyl-_D_-glucoside, _D_-psicose, _D_-raffinose, _L_-rhamnose, _D_-sorbitol, sucrose, _D_-trehalose, turanose, xylitol, acetic acid, *cis*-aconitic acid, citric acid, formic acid, _D_-galactonic acid lactone, _D_-galacturonic acid, _D_-gluconic acid, _D_-glucosaminic acid, _D_-glucuronic acid, *α*-hydroxybutyric acid, *p*-hydroxyphenylacetic acid, itaconic acid, *α*-ketoglutaric acid, *α*-ketobutyric acid, *α*-ketovaleric acid, malonic acid, propionic acid, quinic acid, _D_-saccharic acid, sebacic acid, succinic acid, bromosuccinic acid, succinamic acid, glucuronamide, _L_-alaninamide, _L_-alanyl-glycine, _L_-asparagine, _L_-aspartic acid, glycyl-_L_-aspartic acid, glycyl-_L_-glutamic acid, _L_-histidine, hydroxyl-_L_-proline, _L_-leucine, _L_-ornithine, _L_-phenylalanine, _L_-pyroglutamic acid, _D_-serine, _L_-serine, _L_-threonine, _DL_-carnitine, *γ*-aminobutyric acid, urocanic acid, inosine, uridine, thymidine, phenyethylamine, putrescine, 2-aminoethanol, 2,3-butanediol, glycerol, _DL_-*α*-glycerol, glucose-1-phosphate and glucose-6-phosphate as the sole carbon and energy source. The main cellular fatty acids are C_16:0_, C_16:1_ *ω*7c, C_18:1_ *ω*9c and C_18:1_ *ω*7c. The G+C content of the type strain is 57.6 mol%. The type strain is A3d10^T^ ( =  JCM 19398^T^  =  CIP 110589^T^  =  KMM 7501^T^), isolated from sea water from Port Philip Bay of the Tasman Sea, the Pacific Ocean. The accession number for the whole genome sequence for strain A3d10^T^ is CP007151.

## Supporting Information

File S1 Includes Figures S1–S3 and Tables S1–S3. Figure S1. Neighbour-joining phylogenetic tree showing the taxonomic position of strains R9SW1^T^ and A3d10^T^ according to their 16S rRNA gene sequences. Figure S2. BLAST genome ring (A) and comparison of all proteins in the genomes in terms of the similar composition of the gene families (B) between strains R9SW1^T^, A3d10^T^, *M. adhaerens* HP15^T^ and *M. hydrocarbonoclasticus* ATCC 49840^T^. Figure S3. Scanning electron micrographs of strains (A) R9SW1^T^ and (B) A3d10^T^. Table S1. Genes and the corresponding primer sequences used for the amplification and sequencing. Table S2. Phenotypic characteristics of strains R9SW1^T^, A3d10^T^ and closely related type strains and type species of the genus *Marinobacter*. Table S3. Cellular fatty acids composition of strains R9SW1^T^, A3d10^T^ and closely related type strains and type species of the genus *Marinobacter*.
(DOCX)
