# Supplementary material for: Marinobacter salarius sp. nov. and Marinobacter similis sp. nov., Isolated from Sea Water
Source: PLoS One. 2014 Sep 8;9(9):e106514. doi: 10.1371/journal.pone.0106514 (PMC4157798; doi:10.1371/journal.pone.0106514)
Supplement: File S1 — Includes Figures S1–S3 and Tables S1–S3. Figure S1. Neighbour-joining phylogenetic tree showing the taxonomic position of strains R9SW1T and A3d10T according to their 16S rRNA gene sequences. Figure S2. BLAST genome ring (A) and comparison of all proteins in the genomes in terms of the similar composition of the gene families (B) between strains R9SW1T, A3d10T, M. adhaerens HP15T and M. hydrocarbonoclasticus ATCC 49840T. Figure S3. Scanning electron micrographs of strains (A) R9SW1T and (B) A3d10T. Table S1. Genes and the corresponding primer sequences used for the amplification and sequencing. Table S2. Phenotypic characteristics of strains R9SW1T, A3d10T and closely related type strains and type species of the genus Marinobacter. Table S3. Cellular fatty acids composition of strains R9SW1T, A3d10T and closely related type strains and type species of the genus Marinobacter. (DOCX) [file pone.0106514.s001.docx]

Supporting Information

*Marinobacter salarius* sp. nov. and *Marinobacter similis* sp. nov., isolated from sea water

Hooi Jun Ng^1^, Mario López-Pérez^2^, Hayden K. Webb^1^, Daniela Gomez^1^, Tomoo Sawabe^3^, Jason Ryan^4^, Mikhail Vyssotski^4^, Chantal Bizet^5^, François Malherbe^1^, Valery V. Mikhailov^6^, Russell J. Crawford^1^, Elena P. Ivanova^1^*

^1^Department of Science, Engineering and Technology, Swinburne University of Technology, PO Box 218, Hawthorn, Victoria 3122, Australia

^2^Universidad Miguel Hernandez, Apartado 18 03550 San Juan de Alicante, Spain

^3^Laboratory of Microbiology, Faculty of Fisheries, Hokkaido University, 3-1-1 Minato-cho, Hakodate 041-8611, Japan

^4^Callaghan Innovation, 69 Gracefield Road, Lower Hutt, New Zealand

^5^Collection de 1’Institut Pasteur, Institut Pasteur, 25-28 rue du Dr Roux, 75724 Paris cedex 15, France

^6^G.B. Elyakov Pacific Institute of Bioorganic Chemistry of the Far-Eastern Branch of the Russian Academy of Sciences, 690022, Vladivostok, Pr. 100 Let Vladivostoku, 159, Primorski Krai, Russian Federation

*E-mail: eivanova@swin.edu.au

**Table S1.** Genes and the corresponding primer sequences used for the amplification and sequencing.

| Locus | Primers | Sequence (5' → 3') | *Ta* | Reference |
| --- | --- | --- | --- | --- |
| *rpoD* | rpoD 70F | ACGACTGACCCGGTACGCATGTAYATGMGNGARATGGGNACNGT | 58.0 | Yamamoto and Harayama, 1998 |
|  | rpoD 70Fs | ACGACTGACCCGGTACGCATGTA |  |  |
|  | rpoD 70R | ATAGAAATAACCAGACGTAAGTTNGCYTCNACCATYTCYTTYTT |  |  |
|  | rpoD 70Rs | ATAGAAATAACCAGACGTAAGTT |  |  |
|  |  |  |  |  |
| *gyrB* | UP-1 | GAAGTCATCATGACCGTTCTGCAYGCNGGNGGNAARTTYGA | 60.0 | Yamamoto and Harayama, 1995 |
|  | UP-2r | AGCAGGGTACGGATGTGCGAGCCRTCNACRTCNGCRTCNGTCAT |  |  |
|  | UP-1S | GAAGTCATCATGACCGTTCTGCA |  |  |
|  | UP-2Sr | AGCAGGGTACGGATGTGCGAGCC |  |  |

**Table S2.** Phenotypic characteristics of strains R9SW1^T^, A3d10^T^ and closely related type strains and type species of the genus *Marinobacter*.

| Characteristics | 1 | 2 | 3 | 4 | 5 | 6 | 7 | 8 | 9 | 10 | 11 | 12 | 13 |
| --- | --- | --- | --- | --- | --- | --- | --- | --- | --- | --- | --- | --- | --- |
| Cell length (µm) | 1.9-3.2 | 1.6-2.5 | 1.3-2.1 | 1.8-2.5 | 2.0-4.0 | 1.7-2.4 | 1.5-3.0 | 2.5-3.5 | 1.2-1.8 | 1.6-2.0 | 2.0-2.5 | 1.4-4.0 | 2.0-3.0 |
| Cell width (µm) | 0.40-0.72 | 0.45-0.55 | 0.40-0.45 | 0.3-0.4 | 1.0 | 0.6-0.8 | 0.6-0.9 | 0.3-0.5 | 0.3-0.5 | 0.5-0.8 | 0.6-0.8 | 0.4 | 0.3-0.6 |
| Growth temperature (°C) | 4-40 | 5-40 | 4-40 | 4-42 | 10-45 | 4-45 | 4-45 | 15-40 | 10-45 | 10-37 | 15-42 | 4-42 | 10-45 |
| pH range | 6-9 | 5-10 | 6-9 | ND | 6.5-9.5 | 5.5-10.0 | >5.5 | 5.0-10.0 | 6.0-9.5 | 5.3-9.3 | 5.0-10.0 | 5.0-9.5 | 6-9.5 |
| Salinity range (%, w/v) | 0.5-20 | 1-12 | 0.5-20 | 0.5-18 | 1-20 | 0.5-20 | 1-20 | 1-15 | 0-15 | 1-25 | 0.5-6.0 | 1-15 | 1-20 |
| Nitrate reduction | - | + (-) | + | + | + | - | + | - | + | ND | + | + | + |
| Nitrite reduction | - | + (-) | - | + (-) | - (+) | - | - | - | - | ND | - | + | - (+) |
| Hydrolysis of starch | + | + | - | - | - | - | - | - | + | - | - | - | - |
| Indole production | - | - | - | - | - | - | - | - | ND | ND | + | - | - |
| Fermentation of: |  |  |  |  |  |  |  |  |  |  |  |  |  |
| _D_-Glucose | - | - | w | w | - | w | - | ND | ND | ND | + | + | - |
| Lactose | - | - | - | - | - | w | - | ND | ND | ND | ND | ND | - |
| Acid production from: |  |  |  |  |  |  |  |  |  |  |  |  |  |
| Lactose | - | - | - | - | - | - | - | ND | ND | ND | + | ND | - |
| Raffinose | - | - | - | - | - | - | - | ND | ND | ND | + | ND | - |
| Utilisation of: |  |  |  |  |  |  |  |  |  |  |  |  |  |
| Citrate | - | - (+) | - | - | - | - | - | - | + | - | ND | - | - (+) |
| Dextrin | + | + | - | - | - | w (+) | w | + | + | - | - | ND | - |
| Glycogen | + | + | + | - | - | - | - | + | ND | - | ND | ND | - |
| Tween 80 | + | + | + | + (w) | + | + | + | + | + | + | - | + | + |
| *N*-Acetyl-_D_-glucosamine | - | - | - | - | - | - | - | + | - | - | + | + | - |
| _D_-Cellobiose | - | - | - | - (+) | - | - | - | - | w | - | ND | ND | - |
| _D_-Fructose | + | w (+) | - | - | - | - | - (+) | + | + | - | ND | + | - |
| Maltose | + | w (+) | - | - | - | - | - | + | ND | - | ND | - | - |
| _D_-Mannitol | - | - (v) | - | - | - | - | - | + | - | - | ND | - | - |
| _D_-Sorbitol | - | - | - | - | - | - | - | - | + | - | - | ND | - |
| Sucrose | - | - | - | - | - | - | - | - | + | - | - | ND | - |
| _D_-Trehalose | - | - | - | - | - | - | - | + | + | - | ND | ND | - |
| Mono-methyl-succinate | + | - | + | + (-) | + | + | w | - | ND | - | ND | ND | + |
| Acetic acid | + | + | - | - | - (+) | w | - | - | + | + | + | ND | + |
| _D_-gluconic acid | - | - (+) | - | - | - | - | - (+) | + | ND | - | ND | + | - |
| *β*-Hydroxy-butyric acid | + | + | + | + (-) | + | + | + | - | ND | - | ND | ND | + (-) |
| *γ*-Hydroxy-butyric aicd | + | + | + | - | - | w (+) | w | - | ND | - | ND | ND | - |
| *α*-Keto-butyric acid | - | - | - | - (+) | - | - | - | - | ND | - | ND | ND | - |
| *α*-Keto-valeric acid | - | - | - | - (+) | - | + | - | - | ND | - | ND | ND | + |
| _D,L_-Lactic acid | + | + | + | + (-) | + | + | + | - | - | + | ND | ND | + |
| Propionic acid | + | + | - | - | - | - | - | - | + | - | + | ND | - |
| Succinic acid | + | - (+) | - | - | + | w | - (+) | - | + | - | + | ND | + |
| Bromo succinic acid | - | - (+) | - | - | + | - (+) | - | - | ND | - | ND | ND | + |
| _L_-Alaninamide | w | - | - | - | w | + | - | - | ND | - | ND | ND | - |
| _D_-Alanine | w | + | + | + (-) | w | + | + (-) | - | ND | - | ND | ND | - |
| _L_-Alanine | w | + | + | + (-) | + | + | + (-) | - | + | - | + | ND | w (-) |
| _L_-Glutamic acid | + | + | + | w (-) | + | + | + (-) | - | - | - | + | ND | + |
| _L_-Leucine | w | + | - | - | - | + | - | - | ND | - | ND | ND | - |
| _L_-Phenylalanine | - | + | - | - | - (+) | - | - | - | ND | - | ND | ND | - |
| _L_-Proline | + | + | + | + (-) | + | + | + | - | + | - | ND | ND | + |
| _L_-Serine | + | - | - | - (+) | - | - | - | - | ND | - | ND | ND | - |
| Glycerol | + | - (+) | - | - | w (+) | + (-) | - | - | + | - | + | + | - |
| Enzyme acitvities: |  |  |  |  |  |  |  |  |  |  |  |  |  |
| Lysine decarboxylase | - | - | - | - | - | + | - | - | ND | ND | + | ND | + (-) |
| Ornithine decarboxylase | - | - | - | - | - | - | - | - | ND | ND | + | ND | - |
| Arginine dihydrolase | - | - (+) | - | - | - | - | - | - | ND | ND | + | - | - |
| Esterase (C4) | + | + | + | + (-) | + | + | + | ND | ND | + | w | + | + |
| Lipase (C14) | - | w | w | w (-) | - (+) | w | w (+) | ND | ND | - | - | + | w (-) |
| Valine arylamidase | + | w | w | - | - (w) | - | - | ND | ND | + | - | + | - (w) |
| Cystine arylamidase | + | w | w | - | w | w | - | ND | ND | + | - | + | - |
| Trypsin | - | - | - | - | - | - | - | ND | ND | - | - | + | - |
| *α*-Chymotrypsin | - | - | - | - | - | - | - | ND | ND | - | - | + | - |
| Acid phosphatase | w | w | w | w (-) | w | + | w (+) | - | ND | + | - | + | w (+) |
| Naphthol-AS-BI-phosphohydrolase | w | + | + | + | w (+) | + | w (+) | ND | ND | + | + | + | + |
| ***N***-Acetyl-*β*- glucosaminidase | + | w | + | w (+) | w (+) | w | + | + | ND | + | + | + | + |
| DNA G+C content (mol%) | 57.1 | 55.0 | 57.6 | 56.5 | 55.9 | 56.9 | 58.0 | 57.0 | 57.9 | ND | 57.1 | 57.1 | 52.7 |

Strains: 1, strain R9SW1^T^; 2, *M. algicola* LMG 23835^T^; 3, strain A3d10^T^; 4, *M. sediminum* LMG 23833^T^; 5, *M. salsuginis* CIP 109893^T^; 6, *M. adhaerens* CIP 110141^T^; 7, *M. flavimaris* CIP 108615^T^; 8, *M. lipolyticus* SM19^T^; 9, *M. gudaonensis* SL014B61A^T^; 10, *M. goseongensis* En6^T^; 11, *M. xestospongiae* UST090418-1611^T^; 12, *M. guineae* M3B^T^; 13, *M. hydrocarbonoclasticus* SP.17^T^.

Data for nitrate and nitrite reduction, starch hydrolysis, fermentation, indole and acid production, organic substrates utilisation, and enzyme activities for strains R9SW1^T^, *M. algicola* LMG 23835^T^, A3d10^T^, *M. sediminum* LMG 23833^T^, *M. salsuginis* CIP 109893^T^, *M. adhaerens* CIP 110141^T^, *M. flavimaris* CIP 108615^T^ and *M. hydrocarbonoclasticus* SP. 17^T^ are from this study. The data in brackets are from previously published work [2, 7, 62-70].

+, Positive; -, Negative; w, Weak reaction; ND, no data available.

**Table S3.** Cellular fatty acid composition of strains R9SW1^T^, A3d10^T^and closely related type strains and type species of the genus *Marinobacter*.

| Fatty acids | 1 | 2 | 3 | 4 | 5 | 6 | 7 | 8 | 9 | 10 | 11 | 12 | 13 |
| --- | --- | --- | --- | --- | --- | --- | --- | --- | --- | --- | --- | --- | --- |
| C_12:0_ | 3.6 | 5.2 | 0.6 | 1.7 | 2.0 | 3.0 | 5.5 | 3.5 | 4.7 | 1.2 | 5.0 | 3.4 | ND |
| C_12:0_ 3-OH | 2.0 | 2.1 | 2.8 | 2.0 | 2.6 | 1.9 | 2.1 | 1.9 | 2.0 | 1.9 | 3.8 | 1.5 | 1.7 |
| C_14:0_ | 0.3 | 0.3 | 1.0 | 0.6 | 1.8 | 1.1 | 0.6 | 2.2 | 0.6 | 0.8 | 0.4 | 0.3 | 0.4 |
| C_14:1_*ω*7*c* | 0.6 | 0.3 | 2.2 | 0.9 | <0.2 | <0.2 | 0.3 | <0.2 | <0.2 | <0.2 | <0.2 | 0.5 | 0.2 |
| C_15:0_ | 0.5 | 0.4 | 0.3 | 0.3 | 0.8 | 1.3 | 0.8 | 1.3 | 0.4 | 0.9 | 0.7 | 0.5 | 0.6 |
| C_16:0_ | 29.8 | 26.9 | 15.2 | 22.7 | 38.0 | 27.3 | 27.1 | 38.7 | 31.7 | 29.6 | 27.7 | 25.7 | 17.0 |
| C_16:1_*ω*9*c* | 5.9 | 3.5 | 8.1 | 7.0 | 11.1 | 7.1 | 7.4 | 12.2 | 7.5 | 15.0 | 2.3 | 9.8 | 12.0 |
| C_16:1_*ω*7*c* | 29.1 | 23.1 | 20.9 | 16.5 | 4.6 | 9.0 | 11.2 | 10.7 | 18.0 | 4.7 | 33.7 | 20.8 | 6.5 |
| C_17:0_ | 3.3 | 3.1 | 2.0 | 3.3 | 3.4 | 6.6 | 4.5 | 2.1 | 2.5 | 3.9 | 4.4 | 3.2 | 2.7 |
| C_17:1_*ω*8*c* | 4.4 | 4.4 | 4.9 | 4.7 | 2.0 | 7.5 | 5.9 | 2.5 | 2.8 | 2.3 | 4.8 | 4.0 | 5.1 |
| C_18:0_ | 3.7 | 8.3 | 4.9 | 6.5 | 6.4 | 4.0 | 3.6 | 1.1 | 3.9 | 2.9 | 1.4 | 3.4 | 3.6 |
| C_18:1_*ω*9*c* | 10.6 | 13.5 | 21.2 | 25.4 | 22.7 | 23.2 | 24.5 | 18.9 | 21.7 | 33.9 | 3.0 | 21.3 | 45.3 |
| C_18:1_*ω*7*c* | 12.2 | 19.7 | 14.3 | 8.8 | 1.3 | 1.5 | 1.6 | 1.1 | 2.3 | 0.4 | 9.3 | 3.7 | 4.1 |

All data in the table are from present study. Strains: 1, strain R9SW1^T^; 2, *M. algicola* LMG 23835^T^; 3, strain A3d10^T^; 4, *M. sediminum* LMG 23833^T^; 5, *M. salsuginis* CIP 109893^T^; 6, *M. adhaerens* CIP 110141^T^; 7, *M. flavimaris* CIP 108615^T^; 8, *M. lipolyticus* CIP 107627^T^; 9, *M. gudaonensis* CIP 109534^T^; 10, *M. goseongensis* KCTC 12515^T^; 11, *M. xestospongiae* JCM 17469^T^; 12, *M. guineae* LMG 24048^T^; 13, *M. hydrocarbonoclasticus* SP17^T^ ND, no data available.


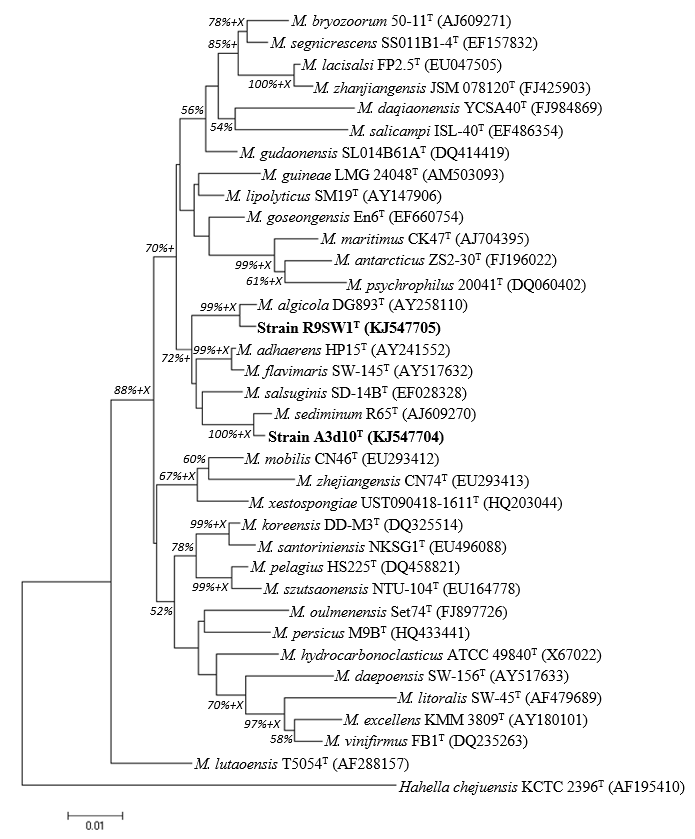


**Figure S1. Neighbour-joining phylogenetic tree showing the taxonomic position of strains R9SW1^T^ and A3d10^T^ according to their 16S rRNA gene sequences.** The sequence of *Hahella chejuensis* KCTC 2396^T^ (AF195410) was used as outgroup. Numbers at branching points are percentage bootstrap values based on 1000 replications, with only values above 50% are shown. Scale bar represents 0.01 substitutions per nucleotide position. The Maximum-likelihood (ML) and maximum Parsimony (MP) algorithms were also used for tree construction, where branches in agreement with ML and MP methods were marked with *+* and *X* respectively.


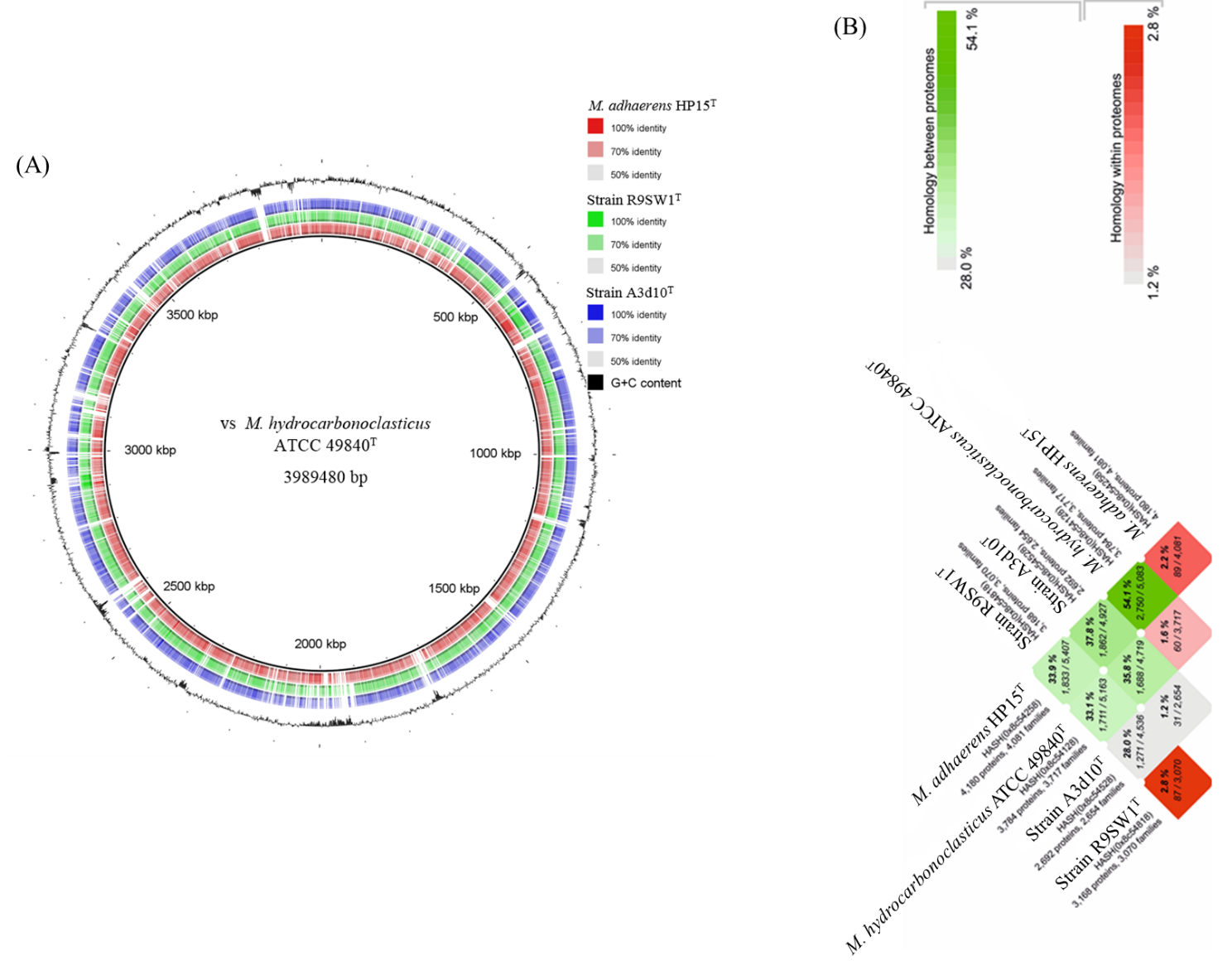


**Figure S2. BLAST genome ring (A) and comparison of all proteins in the genomes in terms of the similar composition of the gene families (B) between strains R9SW1^T^, A3d10^T^, *M. adhaerens* HP15^T^ and *M. hydrocarbonoclasticus* ATCC 49840^T^.**


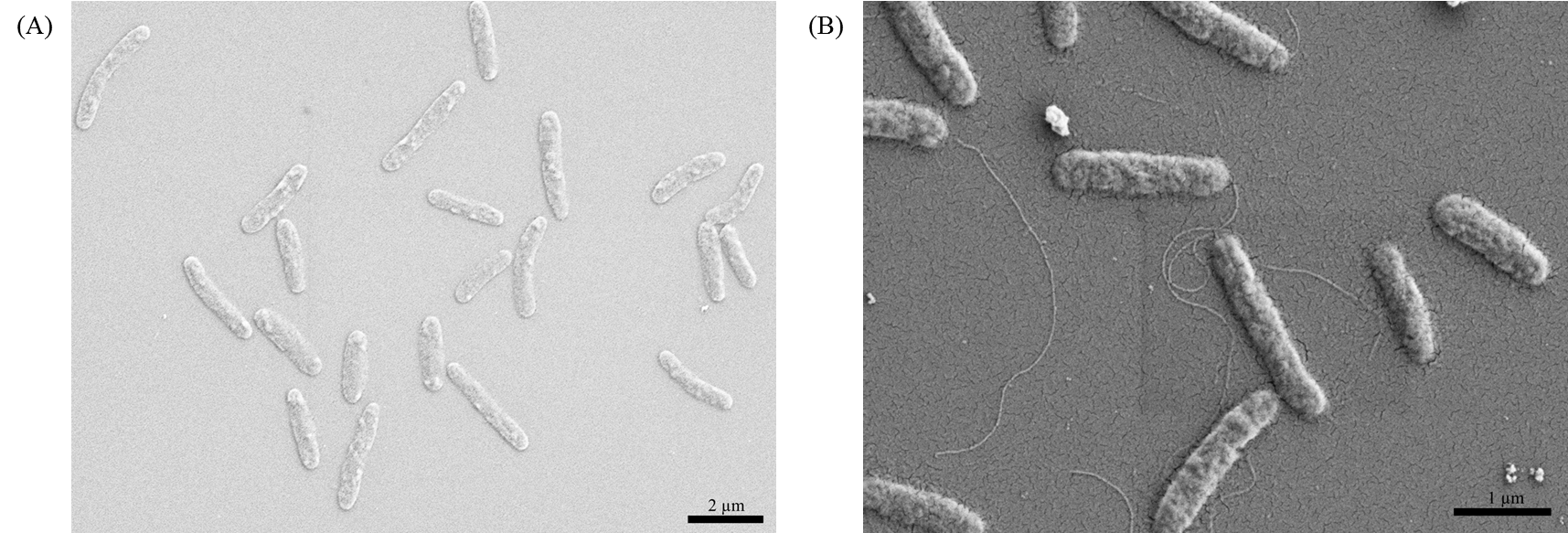


**Figure S3. Scanning electron micrographs of strains (A) R9SW1^T^ and (B) A3d10^T^.**
